# Stress Granules in the Anti-Cancer Medications Mechanism of Action: A Systematic Scoping Review

**DOI:** 10.3389/fonc.2021.797549

**Published:** 2021-12-24

**Authors:** Mohammad Reza Asadi, Marziyeh Sadat Moslehian, Hani Sabaie, Marziye Poornabi, Elham Ghasemi, Mehdi Hassani, Bashdar Mahmud Hussen, Mohammad Taheri, Maryam Rezazadeh

**Affiliations:** ^1^ Student Research Committee, Tabriz University of Medical Sciences, Tabriz, Iran; ^2^ Student Research Committee, School of Medicine, Shahroud University of Medical Science, Shahroud, Iran; ^3^ Department of Molecular Medicine and Biotechnology, Faculty of Medicine, Arak University of Medical Sciences, Arak, Iran; ^4^ Student Research Committee, University of Social Welfare and Rehabilitation Sciences, Tehran, Iran; ^5^ Department of Pharmacognosy, College of Pharmacy, Hawler Medical University, Erbil, Iraq; ^6^ Skull Base Research Center, Loghman Hakim Hospital, Shahid Beheshti University of Medical Sciences, Tehran, Iran; ^7^ Institute of Human Genetics, Jena University Hospital, Jena, Germany; ^8^ Clinical Research Development Unit of Tabriz Valiasr Hospital, Tabriz University of Medical Sciences, Tabriz, Iran

**Keywords:** stress granule, anti-cancer medication, bortezomib, sorafenib, oxaliplatin, 5-fluorouracil, cisplatin, doxorubicin

## Abstract

Stress granule (SG) formation is a well-known cellular mechanism for minimizing stress-related damage and increasing cell survival. In addition to playing a critical role in the stress response, SGs have emerged as critical mediators in human health. It seems logical that SGs play a key role in cancer cell formation, development, and metastasis. Recent studies have shown that many SG components contribute to the anti-cancer medications’ responses through tumor-associated signaling pathways and other mechanisms. SG proteins are known for their involvement in the translation process, control of mRNA stability, and capacity to function in both the cytoplasm and nucleus. The current systematic review aimed to include all research on the impact of SGs on the mechanism of action of anti-cancer medications and was conducted using a six-stage methodological framework and the PRISMA guideline. Prior to October 2021, a systematic search of seven databases for eligible articles was performed. Following the review of the publications, the collected data were subjected to quantitative and qualitative analysis. Notably, Bortezomib, Sorafenib, Oxaliplatin, 5-fluorouracil, Cisplatin, and Doxorubicin accounted for the majority of the medications examined in the studies. Overall, this systematic scoping review attempts to demonstrate and give a complete overview of the function of SGs in the mechanism of action of anti-cancer medications by evaluating all research.

## Introduction

Stress granules (SG) are, from a higher perspective, a subset of RNP granules. Cellular mRNAs appear in the messenger ribonucleoprotein (mRNP) structure within the cell by being coated with proteins (1). SGs are structured, ranging from 100 to 2000 nm, and present in cytoplasmic foci (2). The word stress in the title of these granules indicates the effect of stress on the formation of SGs. Types of stress can be divided into two categories: conditional such as heat shock, arsenite, and hypoxia (3) and other categories including genotoxic drugs and x-ray (4). Stress granules proteins component can be included in three subgroups of RNA binding proteins, non-RNA binding proteins, and transcription initiation factors (5). Proteomic studies and the study of interprotein interactions of this structural component of SGs indicate a large number of proteins that can be included in the structure of SGs (6, 7). Identification of this protein component is essential when more attention is paid to the mechanism of formation of SGs and their effect on the pathogenesis of various diseases. In general, the stress on the cell is followed by the cessation of one of the most critical cell processes called translation (8). Stopping translation accurately at the initiation stage provides the cell with a supply of resources like the RNA-binding proteins involved in this process to build SGs (5).

Interestingly, the major component of the protein component of SGs is RNA-binding proteins that have two specific domains that predispose to the formation of protein aggregates and the construction of SGs, including prion-like domains (PLDs) and intrinsically disordered domains (IDDs) (9). Among these, proteins such as TIA1, PABP, and G3BP have the most involvement in the structure of SGs (10). These proteins can participate in the formation of SGs in two ways. First, the core structure is formed before forming the outer shell structure, where proteins such as G3BP1 and TIA1 attach to the mRNA in the nucleus and form the mRNP structure. This mRNP is transported to the cytoplasm as a core for the SGs formation, although it can also follow the translation process (11, 12). Then, by increasing the core size and connecting other components, a structure of 200 nm is formed, and by continuing the same process, a liquid-like shell is created by relying on microtubules, and it completes the SG structure. Liquid–liquid phase separation (LLPS) is a thermodynamically driven, reversible event that involves the separation of a liquid into two separate liquid phases with differing solute concentrations (13). Alternatively, the structure of SGs can begin to form during the liquid-liquid phase separation process before the formation of the core structure (14) due to poorly binding untranslated mRNPs (15). Then, with the addition of more untranslated mRNPs and more proteins as SGs protein components, the formation of the structure of SGs follows (16).

It should be noted that the structure of SGs is temporary. Cells under stress use SGs as a strategy to protect the translation process, and as the stress is relieved, the structure of SGs moves toward disassembling (17). Disequilibrium between assembly and disassembly can create the conditions for the pathogenesis of various diseases, from neurodegenerative diseases (18, 19) to autoimmune diseases (20) and cancer (21). SGs are involved in various dimensions of cancer, from formation to progression, metastasis, and response to various forms of treatment (22). Cancer is identified by abnormal cell proliferation with the potential to invade and spread to other parts of the body (23).

SGs are present in many cancers, and their up-reg has been proven in many different tumors, including hepatocellular carcinoma (24), sarcoma (25), pancreatic cancer (26), prostate cancer (27, 28), breast cancer (29), malignant glioma (30). Cancer cells are subjected to various stresses due to overgrowth and overuse of nutrients and the effect of various therapies (31). Cancer cells take advantage of the structural ability of SGs under various stresses to survive (32). This research reviews all the studies in the field of cancer treatment in which traces of SGs have been seen in an attempt to review the progress made in targeting SGs in cancer therapy in order to be able to find ways and means of treating cancer.

## Methods

### The Overall Framework of the Review

The strategy in this article was established on the basis proposed by Arksey and O’Malley (2005) (33). Later versions of this strategy were developed by Levac et al. (2010) (34) and Colquhoun et al. (2014) (35). This review follows a 5-step framework, including the following steps: classification of the research question, search strategy, study selection, charting the data, Collating, summarizing, and reporting the results. Consultation is the sixth and final step, which is not covered in this article. During the article’s writing, the Preferred Reporting Items for Systematic Reviews and Meta-Analysis Extension for Scoping Reviews (PRISMA-ScR) Checklist (36) is used to consider and observe two critical aspects clarity and transparency.

### Classification of the Research Question

The main research question that was developed is as follows:

‘What do studies on the involvement of stress granules in anti-cancer drugs and cancer treatments represent?’

‘What are these anti-cancer medications, and what is their functional mechanism?’

Critical studies are considered to be included in general and comprehensive questions.

### Search Strategy

Researchers used PubMed, Scopus, Cochrane, Google Scholar, Embase, Web of Science, and ProQuest to find the articles. The search was not limited by date, language, subject, or type of publication. Review publications were also revised to ensure that related articles were not neglected. For our research on anti-cancer medications and Stress granules, we almost used the following search query: “cancer*” OR “neoplasm*” OR “cyst*” OR “carcinoma*” OR “adenocarcinoma*” OR “neurofibroma*” OR “tumor*” OR “tumour*” OR “malign*” AND “stress granule” OR “stress granules”. Keywords were selected according to background reading and subject headings in PubMed and Embase search engines to have the most coverage on cancer studies. Medical subject heading (MeSH) for the PubMed database and emtree for the Embase database are correctly used in the search. The most recent search was conducted on October 16, 2021. EndNote X8.1 was used to manage the references.

### Study Selection

The publications found during the search were screened for Stress granules involving anti-cancer medications in humans, cell lines, and animal models. Journal articles, conference presentations, erratum, conference abstracts, and reports were among the publications screened. Two reviewers (MRA and MSM) independently completed the screening (first only title and abstract, second full-text). At this point, the article titles and abstracts were reviewed using the inclusion and exclusion criteria listed below.

#### Inclusion Criteria

- Stress granules involved in anti-cancer medications (any cancer) (all human studies, animal studies, cell culture studies)
- Articles in English only
- Original studies

#### Exclusion Criteria

- Research on stress granules in diseases other than cancer
- Languages different from English
- Non-original studies
- Stress granules have been studied using bioinformatics and impractical techniques.

### Charting the Data

Following the completion of the final articles that address the research questions, the data-charting was created to organize the study variables using the following headings: author’s name, year of publication, country, type of study, human samples, animal models, cell lines, SG protein components, methods, major findings, and references. Separately, two reviewers (MRA and MSM) extracted data from articles using charts.

### Collating, Summarizing, and Reporting the Results

A quantitative and qualitative analysis of the publications’ findings, presented in tables and charts, was performed. The quantitative analysis section reviewed a descriptive numerical summary of the studies’ scope, nature, and distribution. In the qualitative analysis section, the presented data were confirmed in light of the broader context proposed by Levac et al. in a narrative review.

## Results

**Figure 1:**
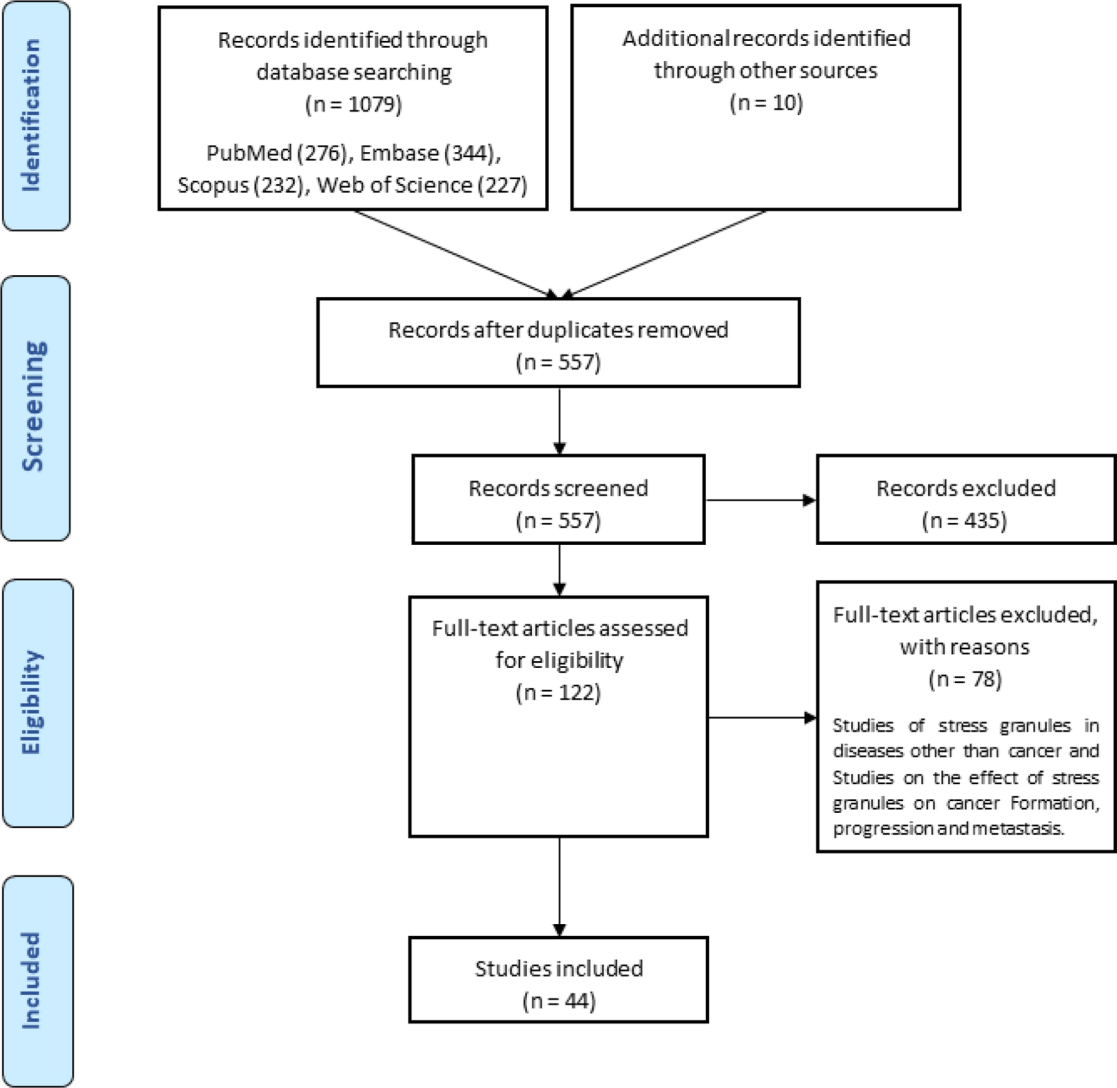
Search strategy flow chart based on the PRISMA flow diagram.

**Figure 2:**
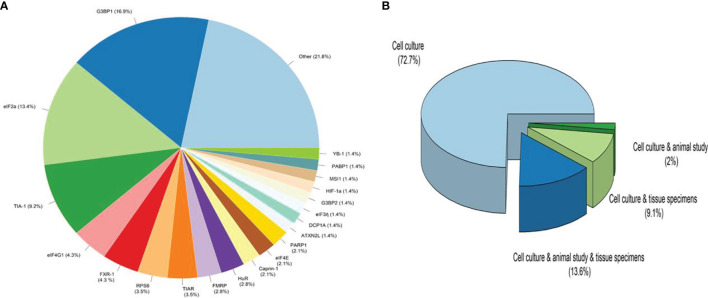
The ratio of Stress Granules protein components and type of studies. **(A)**. Other refers to proteins that have been considered only once in all studies, including CCAR1, DDX3, DDX6, eIF3b, eIF3c, eIF3f, eIF4A1, eIF4D, eIF4E, eIF4G1, FMR1, FMRP, G3BP1, hnRNPA1, hnRNPk, hnRNPA2B1, HSP90a, mTOR, PRMT1, RACK1, RAPTOR, Rbfox2, Sam68, SQSTM1/p62, SRSF1, TDRD3, TIAL1, TTP, USP9X, YWHAZ, ATXN2. **(B)**. Cell culture studies were the most common kind of research, followed by cell culture, animal studies, and tissue specimen studies with the most significant number (13.6 percent in study design), cell culture and tissue specimen studies with 9.1 percent, and cell culture and animal studies with 2% of all studies.

**Figure 3:**
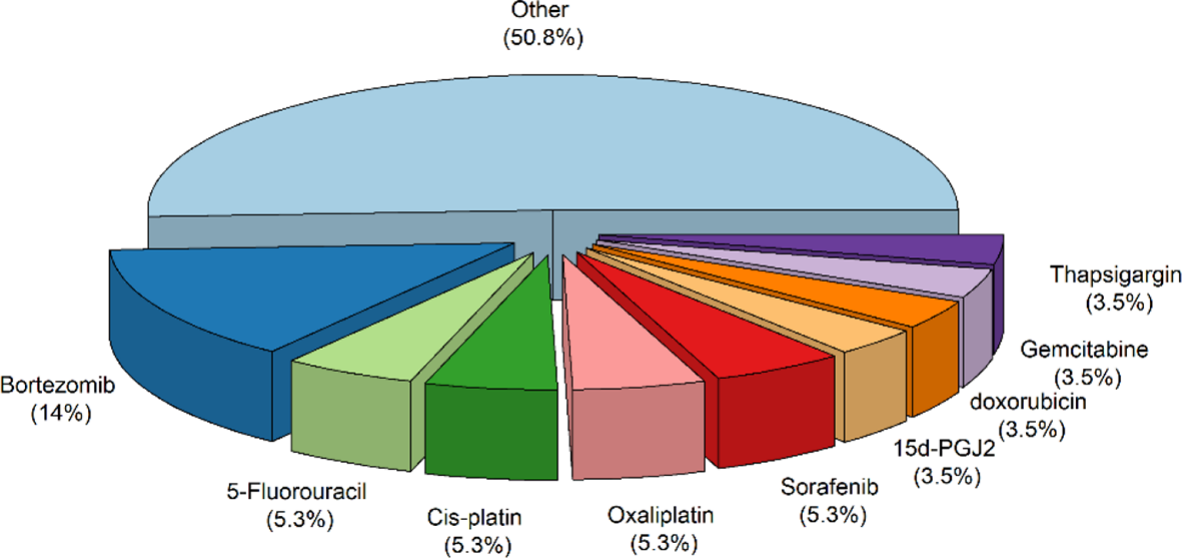
The proportion of anti-cancer medications utilized in studies. Other refers to anti-Cancer medications that have been considered only once in all studies, including Arsenic trioxide, boric acid, c108, camptothecin, Capecitabine, celecoxib, cis-diamminedichloroplatinum, Darinaparsin, docetaxel, fasnall, ibrutinib, Imatinib, lapatinib, Mitoxantrone, Mn3O4, MO-460, morusin, MS-275, nocodazole, Phenethyllisothiocyanate, Psammaplysin F, QLT0267, raloxifene, resveratrol, TAT-RasGAP317–326 (peptide), torkinib, tunicamycin, Verrucarin, Vinca Alkaloid.

A total of ten hundred and seventy-nine items were returned from a keyword search across seven databases. Meanwhile, ten additional records were discovered through other sources, increasing the total number of articles. Endnote software identified and eliminated 522 duplicate records, bringing the total to 557. Following a review of the article titles and abstracts, 122 publications that addressed the research subject were chosen. Following a study of the entire texts of 122 publications, 44 articles for the charting data stage were included in **Table 1**. The procedure for discovering relevant articles and research is depicted in **Figure 1**. Eligible research was published between 2007 and 2021. The percentage of various research is depicted in **Figure 1**. Meanwhile, cell culture research accounts for the vast majority of studies, accounting for approximately 72.7 percent of all studies (24, 30, 37–43, 45, 47, 49, 50, 52, 53, 55, 57–59, 61–63, 65, 66, 68–73, 75, 76). Following that, cell culture, animal, and tissue specimen studies accounted for 13.6% of studies (26, 29, 48, 60, 64, 76), cell culture and tissue specimen studies accounted for 9.1% of studies (46, 51, 54, 67), and cell culture and animal studies accounted for 2% of the total studies (57, 74). Pancreatic cancer (26, 46), gastric cancer (67, 76), breast cancer (29), sarcoma (64), colorectal cancer samples (54, 60), primary malignant B cells (51), and osteosarcoma (48) were among the human cancer samples utilized in the research. **Figure 2** depicts the quantity of each SGs protein component investigated in all investigations. G3BP1 has the greatest rate (16.9%), followed by eIF2α (13.4%), TIA-1 (9.2%), and eIF4G1 and FXR-1 (4.3%). **Figure 3** is a schematic image of the proportion of anti-cancer medications utilized in studies in which bortezomib (26, 30, 39, 42, 59, 62, 63, 73) with 14% has the largest share and followed by 5-Fluorouracil (49, 54, 74), cisplatin (43, 48, 58), Oxaliplatin (26, 67, 76), and Sorafenib (24, 57, 62) with 5.3% of all anti-cancer medications used in studies. The number of studies is limited to twelve countries, with the United States accounting for the most with nine, followed by Canada with eight, China with six, South Korea with four, Switzerland, Germany, Brazil, Japan, and Australia with two each, and Italy, France, Poland, the United Kingdom, Argentina, Chile, and Russia with one each.

**Table 1:** SGs in the mechanism of action of anti-Cancer medications.

Author(s)	Year of publication	country	Type of study	Human sample(s)	Animal model(s)	Cell line(s)	Anti-cancer medications	SGs protein components	Major findings	Refrences
**Kim, W. J. et al.**	**2007**	**South Korea**	**Cell culture**	**-**	**-**	**HeLa cells**	**15d-PGJ2**	**TIA-1** **eIF3b** **eIF3c** **eIF4A1 eIF4E** **HuR** **TIAR** **PABP1** **RPS6**	**15d-PGJ2 has anti-cancer action *via* inhibiting eIF4A, reducing translation, and sequestering TRAF2.**	(37)
**Busa, R. et al.**	**2010**	**Italy**	**Cell culture**	**-**	**-**	**HeLa cells** **PC-3**	**Mitoxantrone**	**Sam68** **TIA-1** **hnRNP A1** **ASF/SF2**	**Sam68 is localized in the structure of SGs within the nucleus as a result of DNA damage.** **Sam68 is up-regulated in prostate cancer and enhances resistance to genotoxic stress.** **Mitoxantrone-induced nuclear stress also impacts CD44 splicing by following the location of sam68 in the structure of SGs.**	(38)
**Fournier, M. J. et al.**	**2010**	**Canada**	**Cell culture**	**-**	**-**	**HeLa cells** **Calu-1** **Hs578T**	**Bortezomib**	**eIF2α** **HuR** **G3BP1** **FMRP** **FXR-1**	**Bortezomib treatment causes the phosphorylation of eIF2α by the Heme Regulated Inhibitor Kinase, which leads to the production of SGs. Bortezomib inhibits HRI, preventing the production of SGs and inducing apoptosis.**	(39)
**Kalra, J. et al.**	**2010**	**Canada**	**cell culture**	**-**	**-**	**Mycoplasma**	**QLT0267**	**YB-1**	**TWIST suppresses YB-1 expression by inhibiting Integrin Linked Kinase (ILK) activity. YB-1 is a protein found in the structure of SGs and controls the expression of Her2 and neu.** **ILK inhibitors may be an excellent way to treat Her2/neu positive cancers.**	(40)
**Annibaldi, A. et al.**	**2011**	**Switzerland**	**cell culture**	**-**	**-**	**U2OS** **HCT116** **HEK293T** **HeLa cells** **CCL39**	**TAT-RasGAP317–326 (peptide)**	**G3BP1** **TIA-1**	**There is a connection and association between G3BP1 and TAT-RasGAP317-326; however, TAT-RasGAP does not sensitize tumor cells to chemotherapy *via* G3BP1.**	(41)
**Gareau, C. et al.**	**2011**	**Canada**	**cell culture**	**-**	**-**	**HeLa cells** **Calu-1** **MCF-7**	**Bortezomib**	**eIF2α** **HuR** **G3BP1** **FMRP** **FXR-1** **eIF4E**	**Bortezomib-induced p21 upregulation can prevent cells from undergoing apoptosis. The stabilization of p21 mRNA accomplishes this by CUGBP1, which is found in SGs. Cell apoptosis is caused by CUGBP1 cell emptying.**	(42)
**Martins et al.**	**2010**	**France**	**cell culture**	**-**	**-**	**U2OS** **HEK293T** **HeLa cells** **CT26**	**cisplatin** **thapsiGargin** **tunicamycin**	**eIF2α**	**Cisplatin did not phosphorylate eIF2α, had no effect on the production of SGs, and did not cause cancer cell death owing to ER stress.** **Cisplatin, when combined with thapsigargin or tunicamycin, has the ability to cause apoptosis and cell death.**	(43)
**Mason, T. A. et al.**	**2011**	**USA**	**cell culture**	**-**	**-**	**HeLa cells** **NIH 3T3 fibroblasts** **ME-SA and MESA/Dx5 cells**	**Darinaparsin** **nocodazole**	**Caprin-1** **CCAR1** **G3BP1**	**darinaparsin directly inhibits microtubule polymerization while being less hazardous than arsenic trioxide (ATO) and nocodazole. Microtubule disintegration increases SG synthesis by inhibiting microtubule formation.**	(44)
**Woldemichael, G. M. et al.**	**2012**	**USA**	**Cell Culture**	**-**	**-**	**786-O** **UOK-121** **RCC4** **UOK-127** **786-O**	**Verrucarin**	**eIF2α** **PARP1** **RPS6**	**Verrucarin induces apoptosis in the cell by blocking translation in its early stages.** **VHL in the structure of SGs interferes with apoptosis in renal cell carcinoma cells by interfering with the verrucarin-induced apoptotic process.**	(45)
**Wen et al.**	**2012**	**USA**	**Cell culture** **tissue specimens**	**pancreatic cancer tissue**	**-**	**Pc-3** **MIAPaCa-2** **HPDE cells** **HFF-1 cells**	**gemcitabine**	**eIF3f** **hnRNP k** **eIF4G1**	**Increased rRNA levels in cancer cells can contribute to cancer progression. By blocking the binding of hnRNP k to rRNA and reducing its levels in non-SG foci cells, eIF3f lowers its protection.** **In chemotherapy, eIF3f knockdown cells are more susceptible to gemcitabine.**	(46)
**Fournier, M. J.** **et al.**	**2013**	**Canada**	**Cell culture**	**-**	**-**	**HeLa cells** **MCF-7** **Hs578T** **N2a**	**torkinib**	**eIF2α** **mTOR** **RAPTOR** **FMRP** **FXR-1** **G3BP1**	**Torkinib (pp242) has the ability to inhibit mTOR or deplete the cell of eIF4E or eIF4G1, therefore inhibiting the production of SGs in cancer cells. As a result, the p21 anti-apoptotic pathway is blocked, and cancer cells become susceptible to chemotherapy and, eventually, death.**	(47)
**Sabile, A. A. et al.**	**2013**	**Switzerland**	**Cell culture** **tissue specimens** **animal study**	**Primary osteosarcoma biopsies of 59 patients**	**mice**	**SaOS-2** **U2OS**	**Cisplatin**	**Caprin-1** **TIA-1**	**Ectopic expression of caprin1 *via* interaction with cyr61 resulted in the production of SGs containing caprin1, which confers resistance to cisplatin-induced apoptosis and substantially increases early tumor development.**	(48)
**Kaehler, C. et al.**	**2014**	**Germany**	**Cell culture**	**-**	**-**	**HeLa cells** **A459** **DU-145** **HEK293T** **HepG2** **RWPE1** **WI38 fibroblasts**	**5-Fluorouracil**	**ATXN2L** **ATXN2** **DCP1a** **DDX6** **eIF2α** **eIF4G1** **G3BP1** **RACK1** **TIAR**	**5-Fluorouracil, by its interaction with ROCK1, causes the development of SGs, which have a high potential for resistance to chemotherapy.**	(49)
**Podszywalow-Bartnicka, P. et al.**	**2014**	**Poland**	**Cell culture**	**-**	**-**	**32D mouse progenitor cells**	**Imatinib**	**TIAR** **HuR**	**ER stress impacts the ARE site in BRCA1 mRNA and can induce down-regulation in BCR-ABL1 leukemia, eventually leading to genomic instability by activating TIAR, which is part of cytoplasmic SGs.** **Imatinib did not diminish HuR expression and very marginally lowered TIAR expression, but it did reduce HuR binding to BRCA1 mRNA, resulting in BRCA1 mRNA separation from SGs.**	(50)
**Yeomans, A. et al.**	**2016**	**UK**	**Cell culture** **tissue specimens**	**Primary malignant B cells**	**-**	**MCF-7**	**Phenethyllisothiocyanate** **ibrutinib**	**eIF2α**	**PEITC slows mRNA translation *via* decreasing mTORC1, boosting eIF2α phosphorylation, and promoting the assembly of SGs.** **PEITC’s chemopreventive and anti-cancer actions are due to its ability to block the mRNA translation pathway.** **PEITC has the potential to improve the effectiveness of ibrutinib as a chemotherapeutic drug.**	(51)
**Adjibade, P. et al.**	**2015**	**Canada**	**Cell culture**	**-**	**-**	**HeLa cells** **MCF-7** **PC-3** **Huh-7** **Hep3B**	**Sorafenib**	**eIF2α** **FMRP** **FXR-1** **eIF4E** **eIF4G1** **G3BP1**	**Sorafenib, PERK, or eIF2α kinase inhibition is known to be the most critical kinase in promoting the development of SGs.** **The PERK-eIF2α-SG pathway has been identified as the primary mechanism of sorafenib resistance in hepatocellular cancer.**	(24)
**Henderson, K. A. et al.**	**2015**	**USA**	**Cell culture**	**-**	**-**	**DU-145**	**boric acid**	**TIA-1** **eIF2α**	**Dietary boron (boric acid) was found to provide health benefits in Du-145 prostate cells *via* promoting the creation of cytoplasmic SGs and the moderate activation of eIF2α and ATF4.**	(52)
**Grabocka, E. et al.**	**2016**	**USA**	**Cell culture** **Animal study** **tissue specimens**	**Six pancreatic adenocarcinomas and three normal** **tissues adjacent to PDACs**	**NCr nude mice**	**DLD1** **HT-29** **NCI-H747** **NCI-H508** **SNUC-1** **MIAPaCa-2** **Panc-1** **AsPC1** **Capan2** **Hs700T** **HEK293T** **HeLa cells**	**Oxaliplatin** **Bortezomib** **15-dPGJ2**	**G3BP1** **eIF4GI**	**The presence of SGs in K-RAS mutant tumor cells is enhanced by increasing the synthesis of 15-d-PGj2 (a lipid compound).** **Stress resistance is increased by up-regulating SGs.**	(26)
**Szaflarski, W. et al.**	**2016**	**USA**	**Cell culture**	**-**	**-**	**U2OS** **MCF-7** **A549** **SiHa** **MEF**	**Vinca Alkaloid**	**eIF2α** **RPS6**	**Vinca Alkaloid (VA) stimulates eIF4E-BP while inactivating eIF2α, resulting in the formation of SGs devoid of particular signaling molecules. VA inhibits the production of SGs, which decreases cancer cell survival and promotes apoptosis.**	(53)
**Vilas-Boas Fde, A. et al.**	**2016**	**Brazil**	**Cell culture**	**-**	**-**	**C6 (rat glioma)** **U87 MG**	**cis-diamminedichloroplatinum** **bortezomib**	**eIF2α** **G3BP1** **FMR1**	**Resistance to chemotherapeutic treatments is caused in glioma cells by the production of SGs *via* eIF2α phosphorylation.** **Inhibition of eIF2α phosphorylation and SG formation results in enhanced susceptibility to chemotherapeutic drugs.**	(30)
**Chiou, G. Y. et al.**	**2017**	**China**	**Cell culture** **tissue specimens**	**CRC one-stage IIA and two stages IIB** **samples and three normal samples**	**-**	**HT-29** **HCT116**	**5-Fluorouracil**	**MSI1** **PABP1** **eIF4E**	**Musashi1 (MSI1) increases the formation of CD44 cancer stem cells and chemotherapy resistance in colorectal cancer by generating musashi1 associated SGs.** **Musashi-1 granules were formed due to 5-FU, and these granules co-localized with G3BP in the SGs structure.**	(54)
**Narayanan, N. et al.**	**2017**	**USA**	**Cell culture**	**-**	**-**	**VMRC-LCD cells** **MDA-MB-231** **HeLa cells** **HEK293T**	**camptothecin**	**TDRD3** **USP9X** **PRMT1** **TIAR** **G3BP1**	**TDRD3 and USP9X are co-localized together in the structure of cytoplasmic SGs. The presence of TDRD3 is required for USP9X.** **TDRD3 knockdown enhances apoptosis and makes breast cancer cells more sensitive to camptothecin during the control of USP9X de-ubiquitination activity.**	(55)
**Chen, H. Y. et al.**	**2018**	**China**	**Cell culture** **Animal study**	**-**	**Nude mice**	**U87 MG** **U251 MG**	**Arsenic trioxide** **doxorubicin**	**MSI1** **eIF2α**	**MSI1 equips cancer stem cells and enhances chemoresistance in glioblastoma cells *via* altering the PKR/eIF2 pathway and generating SGs.**	(56)
**Chen, W. et al.**	**2018**	**China**	**Cell culture**	**-**	**-**	**ACHN** **786-O**	**Sorafenib** **celecoxib**	**eIF2α**	**Sorafenib induces the development of SGs *via* the GCN2/eIF2α pathway and leads to chemotherapy resistance, which is dependent on cox2 expression.** **In chemotherapy-resistant cells, the combination of sorafenib with a cox2 inhibitor (celecoxib) may be beneficial.**	(57)
**Timalsina, S. et al.**	**2018**	**Japan**	**Cell culture**	**-**	**-**	**HeLa cells** **MCF-7** **HCT116** **MDA-MB-468** **panc-1** **RT4** **OVCAR-5**	**Cisplatin**	**G3BP1** **TIA-1** **eIF2α** **eIF4G1**	**β-estradiol - Progesterone and stanolone (EPS) are two medications that can inhibit the development of SGs.** **EPS can partially prevent the formation of SGs by inhibiting PKR rather than PERK.**	(58)
**Bittencourt, L. F. F. et al.**	**2019**	**Brazil**	**Cell culture**	**-**	**-**	**U87 MG**	**bortezomib**	**G3BP1** **TIA-1**	**G3BP1 knock-down inhibits cell formation and increases clearance of SGs, thereby sensitizing bortezomib-resistant u78 glioblastoma cells and increasing apoptosis.**	(59)
**Choi, S. et al.**	**2019**	**South Korea**	**Cell culture** **Animal study** **tissue specimens**	**50 samples of** **human colon cancer**	**C57BL/6 J mice**	**HeLa cells** **B16-F10 cells**	**resveratrol**	**G3BP1** **Rbfox2**	**Rbfox2 in the structure of SGs promotes cell proliferation by influencing and decreasing RB1 expression.** **Resveratrol inhibits Rbfox2 activity on RB1 and decreases cancer expansion by separating Rbfox2 from the structure of SGs.**	(60)
**Shi, Q. et al.**	**2019**	**China**	**Cell culture**	**-**	**-**	**HEK293T** **LNCaP** **22Rv1** **PC-3** **DU-145** **C4–2**	**docetaxel**	**G3BP1** **TIA-1** **Caprin-1** **TTP** **FXR-1** **TIAL1**	**The production of SGs is substantially enhanced in prostate cancer cells, resulting in resistance to cellular stress caused by chemotherapy medicines such as docetaxel.**	(61)
**Christen, K. E. et al.**	**2019**	**Australia**	**Cell culture**	**-**	**-**	**HEK293T** **MCF-7** **T47D** **HeLa cells**	**Bortezomib** **Sorafenib** **Psammaplysin F**	**G3BP1** **TIA-1**	**Psammaplysin F can substantially affect chemotherapy-resistant cancer cells by decreasing phosphorylated eIF2α, reducing the quantity of SGs, and improving the effectiveness of bortezomib and sorafenib.**	(62)
**Comba, A. et al.**	**2019**	**Argentina**	**Cell culture**	**-**	**-**	**MO59K** **LN-229** **T98G**	**bortezomib**	**TIA-1**	**Bortezomib treatment increased arginylated calreticulin (R-CRT) in connection with SGs in the MO59K (apoptosis-resistant) cell line, while in the HOG (apoptosis-sensitive) cell line, SGs production was reduced, and R-CRT exhibits cytoplasmic distribution.** **R-CRT is required for tumor cells to respond to bortezomib therapy.**	(63)
**El-Naggar, A. M. et al.**	**2019**	**Canada**	**Cell culture** **Animal study** **tissue specimens**	**31 humans** **tissues from primary tumors of Ewing sarcoma**	**mice**	**CHLA-10 EWS cell**	**MS-275**	**YB-1** **HIF-1α** **G3BP1**	**MS-275 treatment enhances YB-1 acetylation and lowers deacetylation, prevents the binding of factors such as HIF1a and G3BP1 to its mRNA, suppresses pro-metastatic activity *via* reducing YB-1 translation, and reduces sarcoma metastasis.**	(64)
**Fuentes-Villalobos, F. et al.**	**2019**	**Chile**	**Cell culture**	**-**	**-**	**Tsc2−/− MEF**	**doxorubicin**	**TIA-1** **G3BP1** **RPS6** **eIF3h** **eIF2α**	**Disc1 is an oxidative stress reactor and a cell component involved in maintaining translation levels and cell stability.** **Survival against sodium arsenite therapy is reduced when Disc1 is degraded or overexpressed.**	(65)
**Kashiwagi, S. et al.**	**2019**	**Japan**	**Cell culture**	**-**	**-**	**HeLa cells** **Cos-1** **K562** **Ku812** **TOM-1** **ALL/MIK** **Mycoplasma** **WEHI-3** **Ba/F3-CL1**	**Thapsigargin**	**HSP90a** **DCP1a**	**In the structure of SGs, Bcr-Abl is co-localized. This colocalization is important in granule formation in Bcr-Abl dependent leukemogenesis.** **Thapsigargin therapy results in the development of these SGs, while imatinib, an ABL kinase inhibitor, inhibits the production of these SGs.**	(66)
**Lin, L.** **et al.**	**2019**	**China**	**Cell culture** **tissue specimens**	**119 gastric cancers**	**-**	**SGC-7901** **BGC-823** **MGC80-3** **MKN45** **GES-1**	**oxaliplatin**	**ATXN2L**	**EGF can promote Ataxin-2-like (ATXN2L) as a stress granule regulator in the PI3/AKT signaling pathway, leading to oxaliplatin resistance and eventually increased cell invasion in gastric cancer.**	(67)
**Soung, N. K. et al.**	**2019**	**Korea**	**Cell culture**	**-**	**-**	**Hep3B** **HEK293T**	**MO-460**	**hnRNPA2B1** **HIF-1α**	**MO-460 is a moracin-derived product that generates and accumulates SGs under hypoxic circumstances by binding and inhibiting hnRNPA2B1 and reducing HIF-1α protein production.** **hnRNPA2B1 has been identified as a unique molecular target in hypoxia-induced tumor survival.**	(68)
**Adjibade, P. et al.**	**2020**	**Canada**	**Cell culture**	**-**	**-**	**T47D** **MCF-7** **U2OS**	**lapatinib**	**FMRP** **FXR-1** **G3BP1** **eIF4G1** **eIF2α** **DDX3**	**Lapatinib stimulates the production of SGs *via* eIF2 phosphorylation *via* PERK.** **Cells become susceptible to lapatinib when PERK-SG formation is degraded by PERK depletion.**	(69)
**Amen, T. et al.**	**2020**	**Germany**	**Cell culture**	**-**	**-**	**HEK293T**	**fasnall**	**TIA-1** **G3BP1** **eIF2α**	**fasnall, an anti-tumor agent (fatty acid synthase inhibitor), can stimulate the production of aberrant SGs with high internal mobility and fast turnover.** **Some anti-tumor drugs increase cell viability by increasing the production of SGs.**	(70)
**Attwood, K. M. et al.**	**2020**	**Canada**	**Cell culture**	**-**	**-**	**U251 MG** **HEK293T** **U3024 MG**	**raloxifene**	**G3BP2** **TIAR** **eIF2α** **RPS6** **SQSTM1/p62** **G3BP1**	**Raloxifene is a medication that extends the dissolving period of SGs produced due to hypoxia from 15 minutes to 2 hours.** **When raloxifene and hypoxia are combined, the number of late apoptotic/necrotic cells rises.** **The presence of G3BP1 is required to keep raloxifene’s delayed dissolution of SGs.**	(71)
**Illarionova, N. B. et al.**	**2020**	**Russia**	**Cell culture**	**-**	**-**	**U87 MG** **U251 MG** **FECH15** **NAF1nor**	**Mn3O4**	**eIF3ή** **G3BP1**	**Mn3O4 outperformed the other nanoparticles evaluated for SG efficacy by eIF2α phosphorylation in glioblastoma cells.** **Mn3O4 penetrates the cell within a few minutes and can stay inside intracellular vesicles for up to 24 hours, acting as a Trojan horse in creating SGs.**	(72)
**Lu, X. et al.**	**2020**	**Australia**	**Cell culture**	**-**	**-**	**Vero** **MCF-7** **T47D** **HEK293T** **HeLa cells**	**bortezomib**	**TIA-1** **G3BP1**	**Chikungunya nsP3 is an RNA virus that affects the SG formation process, increasing bortezomib’s cytotoxicity.**	(73)
**Mukhopadhyay, S. et al.**	**2020**	**USA**	**Cell culture** **Animal study**	**-**	**C57bl/6J mice**	**PC-3** **MIApaca-2** **Capan-1** **Panc-1** **SU.86.86** **PK-1**	**Gemcitabine** **5-Fluorouracil** **Capecitabine**	**G3BP1** **eIF4G1**	**K-ras attempts to prevent the formation of SGs, which are markers of chemotherapy resistance, by boosting the networks involved in glutamine metabolism by up-regulating NRF2, a key regulator in the antioxidant network.** **Gemcitabine improves sensitivity to chemotherapy by inhibiting glutamine.**	(74)
**Park, Y. J. et al.**	**2020**	**South Korea**	**Cell culture**	**-**	**-**	**HeLa cells** **ZR75B** **U2OS** **MEF** **HCT116** **PC-3**	**morusin**	**G3BP1** **eIF2α** **PARP1**	**Morusin, although having anti-tumor properties, promotes the development of SGs.** **Morusin activates PKR, which subsequently phosphorylates eIF2α, resulting in the induction of SGs. Morusin can be an effective anti-tumor agent if SGs are suppressed.**	(75)
**Zhang et al.**	**2021**	**USA**	**Cell culture** **tissue specimens** **Animal study**	**47** Human **breast cancer sample**	**FVB/N mice**	**MCa-PSTC** **CT2A**	**c108**	**G3BP2**	**G3BP1 and PD-L1 were shown to be highly co-expressed in cancer tissues.** **G3BP2 knockdown or silencing by c108 also reduced PD-L1 expression due to enhanced mRNA degradation.**	(29)
**Zhao, J. J. et al.**	**2021**	**china**	**Cell culture** **tissue specimens** **Animal study**	**fifty-five gastric cancer patient samples**	**Balb/c nude** **mice**	**MGC80-3** **HGC-27** **SGC-7901** **BGC-823**	**oxaliplatin**	**PARP1** **G3BP1** **YWHAZ** **eIF4D**	**G3BP1 is overexpressed in gastric cancer, where it represents a significant component of SGs. G3BP1 silencing and cell emptying causes apoptosis and enhances susceptibility to chemotherapy.** **G3BP1 interacts strongly with YWAHZ, and patients with G3BP1highYWHAZhigh had the poorest outcomes compared to other patients.**	(76)

## Discussion

### Stress Granules Branch Off From RNP Granules

RNP granules are non-membrane-bound cellular compartments with high protein and RNA concentrations. Nuclear granules like Cajal bodies, paraspeckles, the nucleolus, and cytoplasmic granules like stress granules and processing bodies fall into this category (77, 78). RNP granules are dynamic in nature and rely on RNA for assembly. As a result, the formation of dynamic RNP granules for the concentration of specific cellular components is a strategy that has been conserved across a wide range of organisms and cellular compartments (79). Among these, P-bodies (80) and SGs (81) are two types of cytoplasmic mRNP granules that form from pools of non-translating mRNA. The P-bodies were discovered during research into the localization of proteins associated with the 5′ to 3′ mRNA decay pathway, and the discovery of mRNA decay mediators in these structures led to the initial hypothesis that P-bodies are cellular sites of mRNA decay (82). SGs were named after dense cytoplasmic bodies formed in chicken embryonic fibroblasts when they were stressed in 1988 (83). SGs are dense bodies made up of RNA and proteins that are found in the cytosol when the cells are under stress (84). Ribonucleoproteins appear in response to various stresses, and their number decreases as the stress fades away and is restricted to SGs being disassembled (22).

### SGs in Cancer Treatment

The most challenging aspect of the clinical picture is the use of SGs by cancer cells in response to treatment and chemotherapy. In cancer cells, the equilibrium between assembling and disassembling SGs versus chemotherapy is disrupted, and this imbalance tends to increase the number of SGs. Aside from pathophysiological conditions, numerous studies have linked cancer cell survival to the accumulation of SGs in response to chemotherapy drugs, which can aggravate cancer. EIF2α phosphorylation is the common denominator of the majority of chemotherapeutic agents (85). It is thought that four stress-related kinases phosphorylate eIF2α (17, 18).

Among these are the double-stranded RNA-dependent protein kinase (PKR), PKR-like endoplasmic reticulum kinase (PERK) (86), haem-regulated inhibitor (HRI), and general control nonderepressible 2 (GCN2) (86, 87). Chemotherapy drugs typically stimulate SG accumulation by activating these phosphorylating kinases. Simultaneously, studies show that targeting SGs as anti-stress granule therapy in combination with conventional chemotherapy could provide a new perspective on cancer treatment and has the potential to be recognized as a new treatment through further research.

### Chemotherapy Drug Traces in the Induction of SG Assembly

#### Sorafenib

Sorafenib has shown anti-tumor efficacy in animal models of RCC (88), HCC (89), and DTC (90) by inhibiting tumor proliferation and angiogenicity and promoting tumor death. Although first identified as a Raf inhibitor, it was later shown that sorafenib has several targets, including many protein kinases in the Ras–Raf–MEK–ERK signaling cascade. Sorafenib has the potential to block a variety of oncogenic Ras and Raf mutations, including the BRAF V600E mutant, which is linked to tumor angiogenesis and invasion, as well as the silencing of tumor suppressor genes in a spectrum of cancer types and also inhibits VEGF receptors, platelet-derived growth factor receptor family proteins (PDGFR and Kit), and FMS-related tyrosine kinase 3 (FLT-3) (91), as well as the oncogenic RET kinase (92) and the degradation of the anti-apoptotic myeloid cell leukemia 1 (Mcl-1) protein (93). Sorafenib was approved for use in solid tumors based on these findings. It also reduces the severity of its side effects because it is a potent inhibitor of epoxide hydrolase solution due to the structure of its distributed 1,3-di urea (94).

Pathways can lead to sorafenib-treated cancer cells becoming resistant to the drug. The formation of SGs can be considered as a frontier in resistance to sorafenib treatment. It should be noted that sorafenib produces SGs in a variety of cancer cells, including HeLa (cervix), MCF-7 (breast), PC3, and LnCaP (prostate), with a high degree of potency (80%) (24). Resistance to sorafenib chemotherapy occurs through the pathway in which ATF4 and PERK are involved. Sorafenib induces eIF2α phosphorylation by PERK, and this phosphorylation leads to the formation of SGs. Phosphorylated eIF2α, on the other hand, induces preferential induction of ATF4 expression, which promotes cell death (95). Low ATF4 expression is required for resistance to chemotherapy due to its activity in promoting the expression of antioxidant and chaperone genes that contribute to cell survival and growth (96). On the other hand, under the influence of Sorafenib, PERK mediates the formation of SGs by phosphorylation eIF2α. By capturing ATF4 mRNA, SGs have been shown to minimize expression to the extent necessary for survival and resistance to chemotherapy and increase chemotherapy resistance (24). Sorafenib phosphorylates GCN2 to phosphorylate its downstream protein, eIF2α, promoting cell apoptosis (57). Meanwhile, cox2 protein, which is more expressed in sorafenib-treated cells, inhibits the apoptotic activity of cells with its anti-apoptotic function (97); Cox2 mRNA is localized in the structure of SGs. Combination therapy with sorafenib and celecoxib, which inhibits cox2, is better in chemotherapy-resistant cells than treatment with sorafenib alone (57). It was found that reducing the number of SGs could increase the effectiveness of chemotherapy. Psammaplysin F is a marine sponge-derived metabolite that has the ability to reduce the number of SGs and increase the effectiveness of chemotherapy drugs such as Sorafenib and Bortezomib (62).

#### Bortezomib

Bortezomib is an anti-cancer drug that was made for the first time in 1995, approved by the food and drug administration (FDA) in 2003 to treat multiple myeloma and mantle cell lymphoma (Velcade, PS-341; Millennium Pharmaceuticals, Inc., Cambridge, MA) (98–100). It is a 26S proteasome inhibitor, modified dipeptide boronic acid derived from leucine and phenylalanine. It could inhibit the proteasome reversibly in mammalian cells (101, 102). The proteasome controls protein production and function in normal cells by degrading ubiquitylated proteins and ridding the cell of aberrant or misfolded proteins (103). Clinical and preclinical evidence supports the proteasome’s role in sustaining myeloma cells’ eternal nature, and cell-culture and xenograft data suggest a similar function in solid tumor malignancies. While various processes are believed to be at work, proteasome inhibition may limit the degradation of pro-apoptotic proteins, prompting programmed cell death in cancer cells (99, 104). The 26S proteasome consists of a 20S core complex and 19S regulatory complex, and remarkably, the β-subunits of the 20S core complex have the catalytic function. bortezomib’s binding position is the threonine hydroxyl group in β1-subunit and β5-subunit of the 20S core in the proteasome structure (99, 102). Bortezomib inhibits the chymotrypsin-like activity of the proteasome through the boronic acid group in its binding to the threonine hydroxyl group in the β5-subunit (105, 106).

Bortezomib restraint more than 75% of proteasomes in whole blood samples up to one hour after the dose of bortezomib (99), and additionally, it binds 83% of human plasma proteins (101). Bortezomib has essential activities such as anti-tumor function, growth inhibition, and suppression of apoptosis. On the other hand, bortezomib prevents the progression of the cell cycle in the transition from the G2 phase to the M phase (107) and could influence the NF-κB signaling pathway, leading to anti-apoptotic target genes and expression of anti-apoptotic proteins (108). NOXA is a pro-apoptotic protein that bortezomib provokes in cancer cells (109, 110). Cytochrome P-450 enzymes 3A4, 2D6, 2C19, 2C9, and1A2 are responsible for metabolizing bortezomib through oxidative ways. According to the reports in this metabolization, two isomers from a single metabolite are generated due to bortezomib deboronation, and hydroxylation and deamination occur (101). The metabolization of bortezomib produces more than 30% inactive metabolites (111).

SGs are constituted by provoking bortezomib in cancer cells such as HeLa cells, Calu-I (lung cancer), and Caco (colon cancer) cells, but not all cancer cells like Hs578T breast cancer cells. Under long-term bortezomib (1 M, 10 h) therapy, the synthesis of SGs under stimulation by bortezomib is reversible; therefore, SGs disassemble and partially activate translation; this event occurs independently of eIF2α dephosphorylation. HRI and GCN2 are two kinases responsible for the phosphorylation of eIF2α caused by Bortezomib induction (39). The findings suggest that HRI may promote cancer cell resistance to bortezomib (39, 81). Following HRI reduction, SG formation decreases, and also IF2α phosphorylation is reduced through bortezomib (12). The efficiency of bortezomib was increased by knocking down the HRI in HeLa cells (62). RACK1 or TRAF2 is an apoptotic molecule inactivated by SGs cause to impede cancer cell resistance to bortezomib (39). Flow cytometry analysis shows that cells were treated with bortezomib, which raised the permeability of the plasma membrane. ​Angiogenesis increased in a conforming in vivo model, U87 cells conditioned culture media under bortezomib for 24 hours. Silencing G3BP1 as an SGs protein component might enhance bortezomib-induced apoptosis (59).

There is arginylated calreticulin in the structure of SGs, and it moves to the plasma membrane, where it can regulate cell death in cells treated with bortezomib. Arginylated calreticulin also acts as an apoptosis promoter (63). Bortezomib’s efficacy for solid tumors is inadequate due to resistance to cell death induction (30); nevertheless, insertion of arginylated calreticulin into the plasma membrane of glioma cells treated with bortezomib can initiate the apoptotic pathway (63). Bortezomib’s cytotoxicity would be increased by inhibiting the development of SGs. Chikungunya virus expressed non-structural protein 3 (nsP3), which might impede the development of SGs by inducing G3BP into cytoplasmic foci. Transfecting nsP3 into cancer cells and then treating them with bortezomib might pave the way for a novel strategy for cancer treatment (73). SGs regulate the production of the anti-apoptotic protein p21WAF1/CIP1; Bortezomib promotes the accumulation of p21 mRNA and its translation. p21WAF1/CIP1 and its regulatory protein CUGBP1 inhibit apoptosis in response to bortezomib therapy (42).

#### 5-Fluorouracil

5-Fluorouracil (5-FU) is a uracil and thymine analog used as an antimetabolite and anti-cancer medicine. In the 1950s, researchers observed that rat hepatoma cells utilize pyrimidine uracil to the biosynthesis of nucleic acid, and this finding showed a clear horizon in cancer treatment (112–114). 5-FU is broadly used for treating solid tumors like breast, gastrointestinal system (colon, rectum, anus, esophagus, pancreas, and stomach), head and neck, and ovary (115). The fluorine atom is placed instead of hydrogen of uracil in the 5-FU structure (113). 5-FU inhibits thymidylate synthase (TS), and its metabolites incorporate into RNA and DNA, hence applying its antineoplastic effect (116). TS is the only enzyme that produces *de novo* thymidylate to DNA replication and repair (117). Increasing dUTP could result from TS inhibition and 5-FU metabolite FdUTP might become misincorporated into DNA (118, 119). As a result of these occurrences are DNA strand breaks and cell death (116). Thymidine kinase produces thymidylate from thymidine, so it is a potential salvage pathway TS deficit and provides a mechanism for resistance to 5-FU (120). Dihydropyrimidine dehydrogenase (DPD) is the rate-limiting enzyme in 5-FU catabolism that turns 5-FU to dihydro fluorouracil (DHFU). DHFU is expressed in the liver, and as well as more than 80% of consumed 5-FU is generally catabolized in the liver (121).

The enzymes responsible for metabolizing uracil and thymine could also metabolize 5-FU, and the mechanism of entering 5-FU into the cell is the same as for uracil. 5-FU undergoes intracellular transmutation to active metabolites such as fluorodeoxyuridine monophosphate (FdUMP), fluorodeoxyuridine triphosphate (FdUTP), and fluorouridine triphosphate (FUTP) (122). The 5-FU metabolite integrated into RNA then prevents pre-rRNA maturation into rRNA (123, 124), damages post-transcriptional modification of tRNAs (125, 126), and the assembly and activity of snRNA/protein complexes, resulting in pre-mRNA restraint splicing (127). The suppression of pre-rRNA maturation into rRNA by 5-FU therapy leads to a lack of synthesis of functional ribosomes (128). The incorporation of 5-FU metabolite into RNA is a factor in triggering SGs assembly (49).

There is a stemness gene in neuronal and epithelium cells, namely Musashi-1, which is an RNA-binding protein (129). A study indicated that Musashi-1 has a fundamental role in increasing the extension of CD44+ colorectal cancer stem cells and SG formation. Remarkably, when colorectal cancer cell lines are treated with 5-FU, Musashi-1 leads to SGs formation. Musashi-1 interacted with SGs through its C-terminal region. 5-FU stimulated SGs contained Musashi-1 along with G3BP. The C-terminal of Musashi-1 is critical for SGs formation under the induction of 5-FU. Furthermore, they realized that Musashi-1 causes colorectal cancer drug resistance by forming SGs during 5-FU treatment because Musashi-1 prevents apoptosis in colorectal carcinoma cells *via* the formation of SGs under 5-FU treatment (54). On the other hand, 5-FU could activate PRK (protein kinase RNA-activated), directing to eIF2α phosphorylation (79), thereby forming SG. Based on experiences, 5-FU influences on SGs formation under stress, and SGs become larger. By induction of 5-FU in HeLa cells, SGs include mediator protein RACK1, and disassembly of SGs was affected (49).

#### Cisplatin

Cisplatin is an anti-cancer medication that is useful in the treatment of a variety of malignancies (130). This compound has the chemical formula cl2H6N2pt, which is essentially insoluble in water but soluble in dimethylpropane and N-dimethylformamide (131). M. Peyron discovered and synthesized cisplatin in 1844. Years later, in 1960, Rosenberg demonstrated that platinum electrolytes might halt cell development (132). Despite its anti-cisplatin function, it produces side effects and difficulties in patients, including nephrotoxicity, ototoxicity, myelosuppression, gastrotoxicity, and allergies (133, 134). These cisplatin adverse effects are most likely caused by the substance’s interaction with the N7 position in purine molecules in DNA or by disrupting the fusion of double-stranded or single-stranded DNA molecules (135). Cisplatin is used to treat several malignancies, including ovarian, testicular, and cervical cancers. However, it is essential to note that in these cancers, tumor cells can develop resistance to cisplatin for a variety of reasons, including reduced cisplatin (DDP) levels in the cell, increased glutathione and glutathione S-transferase activity, accumulation of metallothionein’s in the cell, and improved DNA repair (136). There is widespread agreement that cisplatin enters the cell *via* passive transport, which lends credence to the idea that DDP cannot be absorbed completely (137).

One study discovered that Cisplatin therapy results in a lower rate of SG production than predicted. The fraction of cells containing SGs is modest, accounting for 5% of total cells. It is unknown what causes reduced SG production in cisplatin-treated cells; Cisplatin may interfere with SG formation. On the other hand, most cisplatin-induced SGs are likely to be undetectable under a microscope and are distinct from those generated with sodium azide or sodium arsenite (138). However, one study revealed that cisplatin had no effect on SG formation and had no effect on eIF2α. It does not cause ER stress and, when combined with other chemotherapeutic medicines such as ThapsiGargin or tunicamycin, can cause apoptosis in cancer cells (43). Remarkably, another study noted that a primary effect of cisplatin is to prevent the translation from progressing by increasing 4E-BP1 dephosphorylation and eIF2α phosphorylation, respectively. It inhibits the production of SGs in a concentration-and time-dependent way by targeting ribosomes. Cisplatin inhibits translation initiation and promotes cytosolic small ribosomal 40S subunit aggregation to impede ribosome interaction in translation complexes (139). Resistance to cisplatin can result in SGs containing caprin1, one of the components that may be integrated into their structure, and cause chemotherapy resistance, prevent cisplatin-induced apoptosis, and promote tumor development (48).

#### Gemcitabine

Chemotherapy is likely to give significant local control while also prolonging life. However, there is no practical or widely used therapy for advanced or metastatic pancreatic cancer. Gemcitabine, a deoxycytidine nucleoside analog (2′-deoxy-2′,2′-difluorocytidine; dFdC), has demonstrated anti-cancer efficacy against a wide range of malignancies, including pancreatic, lung, and breast cancers. GEM action is dependent on its entrance into cells, where it is immediately phosphorylated by deoxycytidine kinase (DCK), producing monophosphate and diphosphate (dFdCDP) (140, 141). Because of the inhibition of ribonucleotide reductase, diphosphate has an anti-cancer action. Another active GEM metabolite that may be integrated into DNA is the triphosphate metabolite (dFdCTP). The suppression of DNA synthesis is the most significant mode of action of gemcitabine. When dFdCTP is integrated into DNA, it incorporates a single deoxynucleotide, inhibiting chain elongation. This non-terminal location of gemcitabine prevents DNA polymerases from proceeding, a process known as “masked chain termination,” which also prevents gemcitabine removal by DNA repair enzymes (142).

On the other hand, gemcitabine mediates PERK- eIF2α phosphorylation and suppresses translation at the cellular level (1, 143). In response to various stress events, the eukaryotic initiation factor 2 (eIF2) subunit is phosphorylated at serine 51, triggering the Integrated Stress Response (ISR) (144). Resistance to gemcitabine chemotherapy is achieved in this way: Phosphorylated eIF2α (p-eIF2α) significantly reduces translation initiation and total protein synthesis, enabling cellular resources to be conserved. Furthermore, p-eIF2α promotes the preferential translation of specific mRNAs, most notably ATF4, whose overexpression increases the genes’ expression involved in oxidative stress (OS), metabolism, and nutrition absorption (145, 146). Thus, p-eIF2α gene reprogramming helps cells recover from stress-induced damage, increasing apoptosis in response to moderate stress and enabling survival in response to chronic stress (146, 147). In addition to inhibiting translation, phosphorylated eIF2α causes the cell to produce more SGs (15). In the sorafenib treatment, it was established that SGs promote chemotherapy resistance *via* suppressing ATF4 expression (24). Treatment with gemcitabine also maintains ATF4 preferred expression, which may contribute to chemotherapy resistance (26). After gemcitabine therapy, it was discovered in pancreatic epithelial cells that if eIF3f, a component of SGs, is knocked down, the gemcitabine-resistant cell becomes sensitive to this chemotherapeutic agent (46). On the other hand, gemcitabine can improve the sensitivity of other chemotherapy medicines by blocking glutamine metabolism (74).

#### Oxaliplatin

Oxaliplatin is a third-generation cisplatin analog that has demonstrated promising therapeutic results in colon cancer patients resistant to cisplatin. Oxaliplatin is used in combination with other medicines, such as 5-fluorouracil with leucovorin, to achieve response rates of up to 60%, and the inclusion of irinotecan to enhance pancreatic cancer therapy (148). Oxaliplatin has been linked to several different modes of action. Oxaliplatin, like other platinum-based compounds, causes cytotoxicity primarily through DNA damage. Apoptosis in cancer cells can be induced by the development of DNA lesions, the halt of DNA synthesis, the inhibition of RNA synthesis, and the activation of immunologic responses. Oxaliplatin also has synergistic effects with other cytotoxic medicines, although the underlying processes are less well known (149).

Oxaliplatin resistance, like cisplatin resistance, is obtained by a variety of mechanisms, including lower drug uptake and/or greater efflux of the drug, intracellular sequestration, decreased DNA adduct production, improved DNA repair, or increased adduct tolerance, and decreased sensitivity to platinum DNA adducts (150–152). The overall effect of oxaliplatin absorption and outflow is cellular accumulation. The human copper transporter hCTR1, as well as the organic cation transporters OCT1, 2, and 3, can all facilitate oxaliplatin absorption (SLC22A1-3) (153, 154). P-type ATPases, particularly ATP7A and ATP7B, appear to have a functional role in oxaliplatin efflux or sequestration (155, 156). The production of platinum-DNA adducts may be reduced as a result of decreased oxaliplatin transport. Differences in platinum DNA adducts and downstream signaling may explain the activity in colon tumors that are inherently resistant to cisplatin (157).

Resistance to Oxaliplatin may be connected to SGs. ATXN2L, as an SG component, contributed to the recurrence and development of Gastric Cancer (GC), even when treated with Oxaliplatin. ATXN2L expression was increased by EGF and its downstream PI3K/Akt signaling. On the one hand, ATXN2L overexpression aids migration and invasion through EMT. ATXN2L, on the other hand, aiding SGs assembly during oxaliplatin-induced stress. ATXN2L overexpression resulted in intrinsic and acquired oxaliplatin resistance. In turn, oxaliplatin-resistant cell lines expressed more ATXN2L as well as EGF and EGFR. These findings formed a positive feedback loop connecting EGF, ATXN2L, and oxaliplatin resistance because Oxaliplatin had previously been demonstrated to increase PI3K/Akt signaling in a compensatory way. ATXN2L might be utilized as a prognostic and therapeutic target in GC, primarily if oxaliplatin-based chemotherapy is applied (67).

#### Doxorubicin

Doxorubicin is a commonly used anti-cancer medication; typical indications include hematological (such as leukemia and lymphoma, including both Hodgkin’s and non-lymphoma) Hodgkin’s and solid organ malignancies (such as breast cancer, thyroid cancer, sarcoma, osteosarcoma, Kaposi’s sarcoma, and others) (158–160). It is regarded as one of the frontline medicines in many chemotherapy regimens since it is a time-tested anti-cancer agent. Chemotherapeutic regimens, including Doxorubicin (anthracyclines), are superior to regimens that do not contain anthracyclines in studies (161, 162). The two most widely hypothesized and effective mechanisms related to doxorubicin action are damage to cell membrane DNA and other cellular proteins caused by free radical production and intercalation into the cellular DNA, resulting in failure of DNA repair mediated primarily by topoisomerase IIa (163). Doxorubicin is transformed to the unstable intermediate metabolite semiquinone, which is unstable and is converted back to Doxorubicin throughout the process, generating reactive oxygen species (ROS). These free radicals cause extensive cellular damage, including lipid peroxidation, cell membrane degradation, DNA damage, and the induction of apoptosis (164).

One set of genes is responsible for free radical production (NADH dehydrogenase, NO synthase, and xanthine oxidase). In contrast, the other set is responsible for free radical deactivation (NADH dehydrogenase, NO synthase, and xanthine oxidase) (antioxidants, namely glutathione peroxidase, superoxide dismutase, and catalase) (165, 166). According to the second hypothesized mode of action, when Doxorubicin enters the target cell’s nucleus, it intercalates with the host DNA and targets TOP2A (167). TOP2A is in charge of separating entangled DNA, as well as temporarily generating and eventually repairing double-strand DNAs (double-strand breaks [DSB]) (72). Doxorubicin slows the repair process by interfering with the function of TOP2A, resulting in the formation of a significant number of DSBs (168). The presence of DSBs triggers the apoptotic pathway (caspase-dependent) by activating the p53 and FOXO3 genes. The ratio of anti-apoptotic to pro-apoptotic members of the Bcl2 protein family has changed (169). Other suggested modes of action for Doxorubicin include the inhibition of DNA and RNA synthesis as well as the promotion of mitochondrial ROS generation, which triggers the death cascade (163). Furthermore, Doxorubicin has the ability to activate p53, a tumor suppressor that tries to protect cells from specific tumorigenic changes (170).

Although a variety of stressors have been identified as happening in the tumor microenvironment, including local hyperthermia, UV, ionizing radiation exposure, ER stress, oxidative stress, genotoxic stress, and chemo-toxic and inflammatory stress, oxidative stress best depicts the prevalent phenomena surrounding the tumor tissues. Aside from the oxidative stress caused by hypoxia and ATO treatments, Doxorubicin significantly increases ER stress and pro-apoptotic processes that promote the development of SGs (1). In particular, Doxorubicin increases the number of SGs by directly affecting phosphorylation on eIF2α (53). In a study on the fission yeast Schizosaccharomyces pombe, the effect of Doxorubicin on SGs was further studied, and it was found that in this Schizosaccharomyces pombe, Doxorubicin, along with heat, causes the formation of SGs from a non- eIF2α -independent pathway but is targeted. The formation of SGs decreases the sensitivity of cells to Doxorubicin (171).

## Conclusion

**Figure 4:**
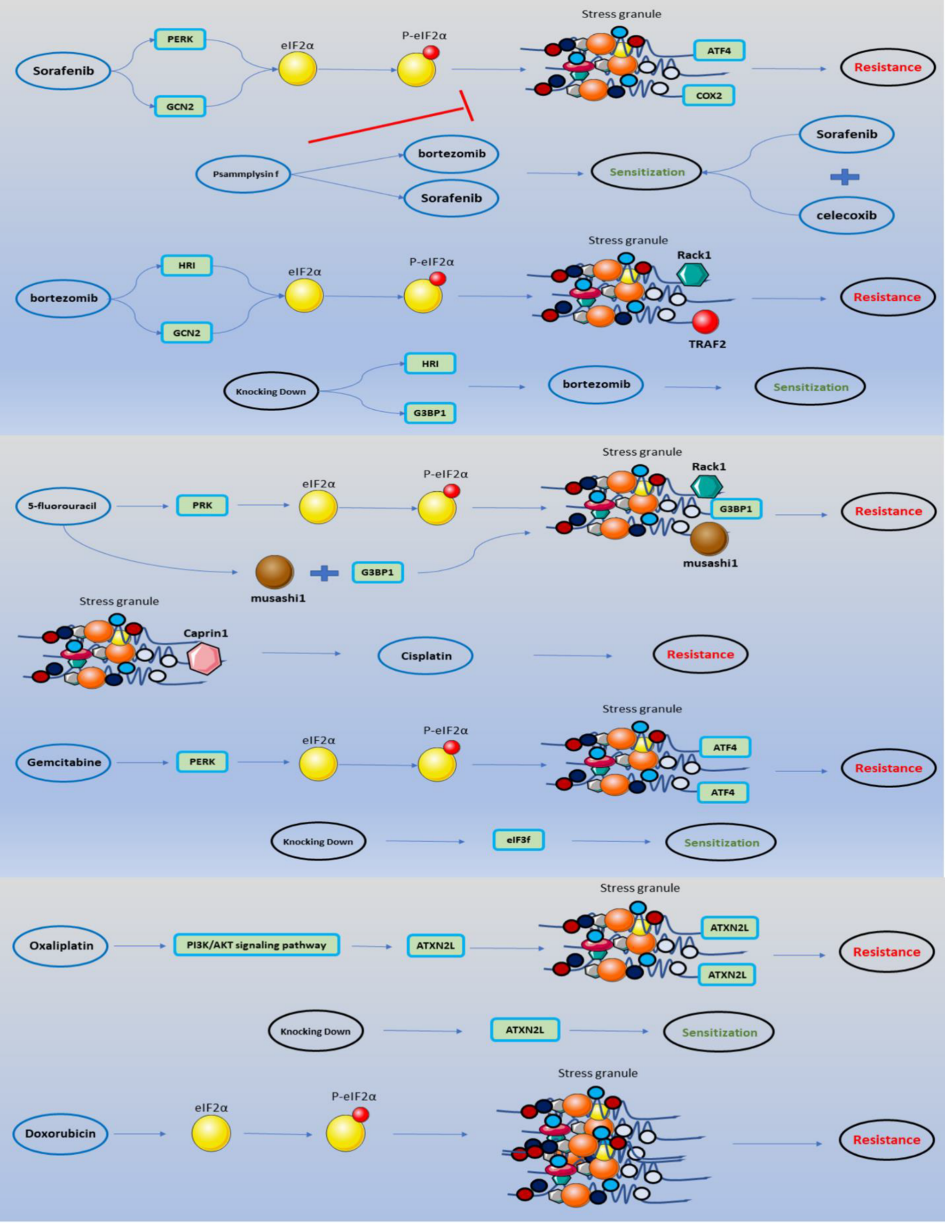
SGs involved in anti-cancer medications mechanism of actions. The impact of anti-cancer medications on the development of SGs through eIF2α phosphorylation is depicted in a schematic. Accumulation of SGs with particular features leads to chemoresistance, which may be anticipated by enhancing the sensitivity of specific medications by combining specific pharmaceuticals or knocking down a portion of the protein components of SGs.

SGs have evolved into one of cancer cells’ primary stress-response mechanisms. SGs allow cancer cells to go through the most challenging phases of their development process on account of their structural capabilities. Many studies have shown that SGs have a role in cancer treatment and responsiveness to anti-cancer medications. A general point of agreement is that SGs are involved in and play a critical role in various pathways in various malignancies. On the other hand, the impact of SGs on cell cycle regulatory factors and critical elements implicated in cancer cell proliferation is utilized as a biased mechanism. Utilizing the capabilities of SGs in the process of chemotherapy resistance (**Figure 4**), as well as the existence of more SGs in cells receiving chemotherapeutic drugs, is associated with cancer at the following critical stages. Much research has been conducted on the effects of SGs on anti-cancer medications. The goal of this research was to offer a comprehensive review to conclude this subject. Overall, this research may pave the way for future investigations on SGs in treating malignancies and offer a roadmap to lead these studies.

## Author Contributions

MA, HS, and MR wrote the draft and revised it. MT designed and supervised the study. BH, MM, MP, EG, and MH contributed in data collection and designing the tables and figures. All authors contributed to the article and approved the submitted version.

## Funding

The research protocol was approved and supported by a grant (grant number: 68047) from Student Research Committee, Tabriz University of Medical Sciences.

## Conflict of Interest

The authors declare that the research was conducted in the absence of any commercial or financial relationships that could be construed as a potential conflict of interest.

## Publisher’s Note

All claims expressed in this article are solely those of the authors and do not necessarily represent those of their affiliated organizations, or those of the publisher, the editors and the reviewers. Any product that may be evaluated in this article, or claim that may be made by its manufacturer, is not guaranteed or endorsed by the publisher.
